# Development and Evaluation of a Questionnaire for Measuring Suboptimal Health Status in Urban Chinese

**DOI:** 10.2188/jea.JE20080086

**Published:** 2009-11-05

**Authors:** Yu-Xiang Yan, You-Qin Liu, Man Li, Pei-Feng Hu, Ai-Min Guo, Xing-Hua Yang, Jing-Jun Qiu, Shan-Shan Yang, Jian Shen, Li-Ping Zhang, Wei Wang

**Affiliations:** 1 School of Public Health and Family Medicine, Capital Medical University, Beijing, China; 2 Physical Examination Center, Beijing Xuanwu Hospital, Capital Medical University, Beijing, China; 3 Multicampus Program in Geriatric Medicine and Gerontology, UCLA School of Medicine, Los Angeles, CA, United States; 4 College of Life Sciences, Graduate University, Chinese Academy of Sciences, Beijing, China; 5 Curtin Health Innovation Research Institute, Curtin University of Technology, Perth, Australia

**Keywords:** suboptimal health status, questionnaire, reliability, validity

## Abstract

**Background:**

Suboptimal health status (SHS) is characterized by ambiguous health complaints, general weakness, and lack of vitality, and has become a new public health challenge in China. It is believed to be a subclinical, reversible stage of chronic disease. Studies of intervention and prognosis for SHS are expected to become increasingly important. Consequently, a reliable and valid instrument to assess SHS is essential. We developed and evaluated a questionnaire for measuring SHS in urban Chinese.

**Methods:**

Focus group discussions and a literature review provided the basis for the development of the questionnaire. Questionnaire validity and reliability were evaluated in a small pilot study and in a larger cross-sectional study of 3000 individuals. Analyses included tests for reliability and internal consistency, exploratory and confirmatory factor analysis, and tests for discriminative ability and convergent validity.

**Results:**

The final questionnaire included 25 items on SHS (SHSQ-25), and encompassed 5 subscales: fatigue, the cardiovascular system, the digestive tract, the immune system, and mental status. Overall, 2799 of 3000 participants completed the questionnaire (93.3%). Test-retest reliability coefficients of individual items ranged from 0.89 to 0.98. Item-subscale correlations ranged from 0.51 to 0.72, and Cronbach’s α was 0.70 or higher for all subscales. Factor analysis established 5 distinct domains, as conceptualized in our model. One-way ANOVA showed statistically significant differences in scale scores between 3 occupation groups; these included total scores and subscores (*P* < 0.01). The correlation between the SHS scores and experienced stress was statistically significant (*r* = 0.57, *P* < 0.001).

**Conclusions:**

The SHSQ-25 is a reliable and valid instrument for measuring sub-health status in urban Chinese.

## INTRODUCTION

A surprisingly large number of city dwellers in China suffer from poor health and many die prematurely.^1^ In addition, there has been an increase in the number of people who report suboptimal health in the absence of a diagnosable condition, which is known as suboptimal health status (SHS) in China. SHS is a physical state between health and disease, and is characterized by the perception of health complaints, general weakness, and low energy.^2^ It is regarded as a subclinical, reversible stage of chronic disease.

In China, chronic noncommunicable diseases now account for an estimated 80% of total deaths and 70% of the total number of disability-adjusted life-years (DALYs).^3^ The major causes of death in China are cerebrovascular disease, cancer, heart disease, and chronic respiratory disease.^4^ Among middle-aged people, rates of death from chronic disease are higher in China than those in some high-income countries.^3^ In 2004, the prevalences of hypertension, coronary heart disease, and diabetes in city-dwelling people aged 15 years or older increased to 18.3%, 6.3%, and 4.5%, respectively.^5^

In addition to the ageing of the population, China is experiencing dramatic transformations in social and economic conditions, and these changes will continue to increase the incidences of major chronic diseases.^6^ From 1990 to 2000, the proportion of people living in urban settings in China increased from 26% to 36%, and the number of cities increased to 663.^7^ It is expected that urbanization in China will reach 45% by 2010, with an extra 200 million residents expected in the urban areas by 2010.^8^ The rapid environmental changes that accompany urbanization are increasing the prevalence of the major risk factors for chronic disease,^6^ including work stress, physical inactivity, unhealthy diet, and tobacco use.

In our initial data analysis, stress was among the most often mentioned factors influencing general health. As China has entered the World Trade Organization (WTO), employees are becoming more frequently exposed to stressful situations^9^ such as overwork, competition, and perceived isolation. Continuous psychosocial stress seems to be a part of the everyday reality for Chinese, especially for white-collar workers.^1^ Several studies have identified stress as a key factor contributing to poor public health.^10^^,^^11^ In a recent 3-year follow-up study, stepwise logistic regression analyses revealed that stress was the factor most often associated with frequent reports of pain and perceived health complaints.^12^

Stress does not always directly result from the stressor itself, but rather from the perception of its effects.^13^ Nowadays, it is understood that psychological strain can provoke important stress reactions.^10^ Acute stress responses promote adaptation and survival via responses from the neural, cardiovascular, autonomic, immune, and metabolic systems. Chronic stress can promote and exacerbate pathophysiology via the same systems that are dysregulated.^14^ Chronic overwhelming stress leads to exhaustion, and this state of exhaustion is marked by energy depletion and tissue degeneration.^15^ Chronic activation of the stress response is an important public health issue from both health and cost perspectives. It is linked to a myriad of health disorders, including diseases of the cardiovascular, gastrointestinal, immune, and neurologic systems, as well as to depression and sleep disorders.^16^

SHS has become a new public health challenge in China. Unfortunately, studies on improving the management of SHS in China are limited. However, with increasing economic development, the prevalence of SHS is expected to escalate. Studies on intervention and prognosis for SHS are expected to become increasingly important. Consequently, the existence of a reliable and valid instrument to assess SHS will be essential. Therefore, we developed and validated a comprehensive questionnaire SHSQ-25 to assess SHS among urban Chinese.

## STUDY 1: QUESTIONNAIRE DEVELOPMENT

### Methods

The phases of questionnaire development included a literature review, focus group discussions, construction of items, a pilot study, and a main study to investigate preliminary validity and reliability.

Focus group discussions were held with ostensibly healthy persons who had recently complained of health problems at 2 hospitals in 2007. Each focus group consisted of 4 to 6 participants, and included both men and women. Focus group discussions were led by a moderator, who adhered to a question guide with 3 categories of questions: social-psychological stress, unhealthy lifestyles, and general health status. The discussions were recorded, transcribed verbatim, processed as text until no new themes emerged, and then analyzed by means of qualitative content analysis.

The item pool for the questionnaire was generated from the themes pertaining to perceived health complaints that were discussed in the focus groups. In addition, we conducted a literature review and solicited expert opinion (provided by a panel comprising 2 health managers, 1 physician, 1 pathophysiologist, and 1 clinical epidemiologist). After the items were written, focus groups were used again to discuss whether the items were relevant, clear, unambiguous, and written in language that would be understandable to potential respondents. Items with an endorsement rate lower than 0.2 were discarded.

### Results

The focus group discussions were not initially centered on stress, but encompassed themes related to health and well-being, such as the participants’ own state of health and the reasons for poor health in urban Chinese. From the onset of the analysis, we attempted to determine how frequently the participants mentioned stress and identified it as one of the most significant causes of impaired health, regardless of whether the discussion of stress was initiated by the moderator. Out of 21 participants, 16 initiated a discussion of stress using the word “stress,” and 2 did so by using related expressions (nervous system, nervous tension). The other 3 participants discussed stress after it was initiated by the moderator. We also noticed that the participants used the word stress both to denote a felt negative psychoemotional state and to identify the reasons for these feelings (stressors, such as competition at work, highly demanding tasks, overwork, and poor personal relationships).

All the participants identified “stress” as being harmful to health. The most frequent symptoms were fatigue (90.5%), headache (52.4%), and discomfort in the back (66.7%), joints (66.7%), and arms or legs (61.9%). Participants also frequently reported mental symptoms such as nervousness (81.0%), poor sleep (42.9%), and cognitive impairment (76.2%); cardiovascular complaints such as heart palpitations (28.6%), pressure in the chest region (28.6%), sweating (23.8%), and breathlessness without exertion (33.3%); gastrointestinal complaints such as poor appetite (52.4%), abdominal pain (28.5%), bloating (23.8%), and food intolerance (23.8%); and common cold symptoms such as cold intolerance (23.8%) and sore throat (33.3%).

One way to understand the effects of stress on health, as the participants’ experience suggests, is to see it as a sociopsychological factor leading to unhealthy behaviors, such as lack of physical activity, inadequate sleep, and unhealthy diet. Stress is also a putative cause of increased smoking and alcohol consumption among men.

The item pool for the questionnaire was generated from the 37 descriptions of health complaints gathered in the discussion. A literature review revealed only a small number of studies that measured SHS. However, several relevant items were derived from a questionnaire study of SHS in 3 occupational groups^17^ (7 items) and the Cornell Medical Index (CMI)^18^ (9 items), which have been proven to be useful and valid. An initial version of the questionnaire was constructed and consisted of 42 items. Items were reviewed for content validity by 4 physicians experienced in health management, all of whom indicated that the questions were relevant. In August 2007, the questionnaire was tested in a pilot study on physical examination centers conducted in 3 hospitals affiliated with Capital Medical University. A total of 30 questionnaires were distributed at the 3 hospitals to persons with diminished well-being, but without a diagnosis of a relevant disease or disorder. All 30 questionnaires were completed and returned to the research unit. Two final items in the pilot questionnaire asked “whether the questions seem relevant” and “whether the questionnaire is too long.” Twenty-nine of the 30 respondents felt that the questions were relevant, and only 2 respondents felt that the questionnaire was too long. The questionnaire was therefore deemed feasible for use in a larger study.

Based on the feedback and the need for brevity, 4 items were dropped from the initial list, which resulted in 38 items. The first 7 items were background/demographic questions. The 25 items related to SHS were categorized into 5 subscales: fatigue (9 items), cardiovascular system (3 items), digestive tract (3 items), immune system (3 items), and mental status (7 items). All questions asked the individual to rate a specific statement on a 5-point Likert-type scale, based on how often they suffered various specific complaints in the preceding 3 months: (1) never or almost never, (2) occasionally, (3) often, (4) very often, and (5) always. Furthermore, 6 questions about stressful situations were included, and were rated on the same scale. A final open question inquired as to any additional feelings regarding the respondent’s health, bringing the total to 39 items. With the exception of the 16 items derived from other questionnaires, all questions originated from the focus group discussions.

### Discussion

The aim of Study 1 was to develop a questionnaire for measuring SHS, an early reversible stage of chronic diseases such as cardiovascular and metabolic diseases. We established an instrument (SHSQ-25), including 25 items on SHS and encompassing 5 subscales of perceived health complaints that were affected by chronic stress.

The concept of stress is relatively new in China, and it may have been more readily expressed by educated people living in cities. In the focus group discussions, stress was mentioned by nearly all 21 participants as a potential cause of physical illness, as was anxiety. However, most participants had difficulty identifying actual diseases caused by stress and in explicating the links between stress and physical illness.

Stressors can produce different responses in different persons or in the same person at different times.^15^ The role of the integrated response of multiple systems is thought to be more important than those of isolated reflexes. Although virtually all organs are affected by stress, the neuroendocrine and immune systems are the first to experience functional changes. Neuroendocrine responses interact with the immune system and affect the cardiovascular, gastrointestinal, reproductive, and other body systems.^19^

Content validity is a measure of the extent to which an instrument encompasses the relevant aspects and attributes of the concepts it aims to measure; the relevance of these aspects is based on the judgments of expert and lay groups.^20^ Content validity in this study was established by means of focus group discussions, literature searches, a review of the questionnaire by experts, and a pilot study. It is acknowledged that patients and potential research subjects are an excellent source of items in scale development.^21^ In the present study, the participants in the focus group discussions were selected from the target group, ie, people with SHS. A total of 16 items from 2 measures of general health status, whose validity had been previously established, were included in the SHS questionnaire (SHSQ) to assist in establishing validity. The pilot study was conducted among a population similar to the focus group, which had reported that the questionnaire encompassed subjects that they deemed relevant. The SHSQ adequately encompassed the domains under investigation and had a sufficient number of items. The reliability and validity of the instrument were further examined in Study 2.

## STUDY 2: DETERMINING THE VALIDITY OF THE QUESTIONNAIRE

### Methods

#### Study participants

Persons undergoing a regular physical examination were recruited at the physical examination center of Beijing Xuanwu Hospital, a teaching hospital affiliated with Capital Medical University. Participants had to meet the following inclusion criteria: (1) no history of somatic or psychiatric abnormalities, as confirmed by their medical records, (2) age from 18 through 60 years, and (3) no history of medication consumption in the previous 2 weeks. All participants underwent a standardized examination, including medical history, physical examination, blood hematology and biochemistry analysis, rest electrocardiography, and abdominal ultrasonography. We excluded individuals with diabetes or a diagnosis of a specific disease of the cardiovascular system, respiratory system, genitourinary system, digestive system, or circulatory system. Subjects with diseases not causally related to their health complaints, as determined by physicians, were included in the study. To ensure comparability of the findings, all participants were examined by physicians specially trained for the study.

Both the hospital and university research ethical committees approved the study, and written informed consent was obtained from all participants before they answered the questionnaire. The procedures for protecting participant privacy were described in the questionnaire.

#### Data collection

The questionnaire was administered after each participant completed the examination at the physical examination center. The participants completed the questionnaire themselves, after instructions were given by a researcher. The completed questionnaire was checked by a researcher to ensure that all questions had been answered. A randomly selected 5% of the participants were asked to complete the same questionnaire again exactly 7 days later, when they returned to the hospital to learn the results of their physical examination.

#### Statistical analyses

Before analysis, all questionnaires were reread and checked for accuracy. For data processing and analysis, SPSS v11.5 was used. The raw scores of 1 to 5 on the questionnaire were recoded as 0 to 4. SHS scores were calculated for each respondent by totaling the ratings for the component subscale items. The score for each subscale was then transformed linearly to a scale ranging from 0 to 100 points, with 0 indicating the lowest level of SHS (good health) and 100 indicating the highest level (poor health). The participants’ scores for their experienced stresses were also converted to a 100-point scale. The reliability and validity of the 25-item SHSQ were assessed using the methods described below.

##### Test-retest reliability.

The test-retest reliability between the first and second measurements was calculated for all individual items by using ICCs (intraclass correlations),^22^ which were subsequently used to calculate the mean overall test-retest reliability and the mean subscale reliability (type of symptoms). An ICC lower than 0.50 was defined as poor reliability, 0.50 to 0.75 as moderate reliability, and higher than 0.75 as good reliability.^22^

##### Internal consistency.

Internal consistency is a measure of reliability that assesses the degree to which the items are related to each other; it measures a unified construct.^23^ Item-internal consistency (IIC) was assessed by correlating each item score with its dimension score, which was corrected for overlap. A correlation of 0.40 has been suggested as the standard for supporting an IIC.^24^ Then, as a measure of the overall consistency of items, Cronbach’s α was determined for each subscale. A Cronbach’s α coefficient of 0.70 or higher is considered satisfactory.^25^

##### Factor structure.

Bartlett's test of sphericity and the Kaiser-Meyer-Olkin measure of sampling adequacy (KMO) were used to assess the factorability of the correlation matrix. KMO values should be close to 1, and a minimum value of 0.6 has been suggested.^26^ Maximum likelihood (ML) factors analysis with subsequent promax rotation was used to extract the factors.^27^ A factor loading of at least 0.37 was used to determine cut-off points.^28^ The multidimensional structure of the instrument was further assessed by confirmatory factor analysis (CFA), using SAS 8.2. The ML method was used to test the goodness of fit by several indices, namely, χ^2^, the root mean square error of approximation (RMSEA), the goodness-of-fit index (GFI), and the adjusted goodness-of-fit index (AGFI). RMSEA values lower than 0.08, and GFI and AGFI values greater than 0.90, indicated reasonable fit.^29^^,^^30^

##### Discriminative ability.

Statistically significant differences in scale scores between occupations or age groups would offer evidence of the questionnaire's ability to discriminate between groups. According to our conceptual model, the study attributed deteriorating health to chronic social-psychological stress caused mainly by working conditions. We therefore expected statistically significant differences between the white-collar workers, blue-collar workers, and college students. Statistically significant differences in scale scores were also expected between different age groups.^31^ One-way analysis of variance (ANOVA) was used to compare the scale scores among different groups. Post-hoc pair comparisons were performed using the least-significant-difference (LSD) test.

##### Convergent validity.

Convergent validity was evaluated by examining the extent to which certain scales correlated to theoretically related variables.^28^ We hypothesized that the SHS scores would significantly correlate with the scores for psychosocial stress.

### Results

#### Characteristics of participants

A total of 3000 questionnaires were distributed during the 8-month study period; 2799 completed responses were received (93.3% response rate). Table 1 shows the characteristics of participants. Mean age was 42.2 years (SD, 11.9) and 56.4% were women. Majorities of respondents had a university degree (63.4%) and were white-collar workers (53.5%).

**Table 1.** Characteristics of Questionnaire Respondents (*n* = 2799)

Variable	*n*	%
Sex		
​ Female	1580	56.4
​ Male	1219	43.6
Age (yrs)		
​ 18–30	613	21.9
​ 31–40	881	31.5
​ 41–50	834	29.8
​ 51–60	471	16.8
Highest level of education		
​ Compulsory education (through grade 9)	192	6.9
​ High school graduation	831	29.7
​ University/college degree	1776	63.4
Occupation		
​ White-collar worker	1497	53.5
​ Blue-collar worker	507	18.1
​ College student	345	12.3
​ Other	450	16.1

#### Test-retest reliability

Of the 150 respondents included in the test-retest study, 143 completed the second questionnaire (95.3%), which was given 7 days after the first questionnaire. There were 67 (46.9%) men and 76 (53.1%) women, and their mean age was 41.9 years (SD, 9.6). The median ICC between the original and retest ratings for individual items was 0.94; the ICC ranged from 0.89 to 0.98. The ICC for the overall SHS and domain ICCs were good for the sample (Table 2).

**Table 2.** Results for 5 Subscales of the 25-item SHSQ

Subscale	No. of items	Mean ± SD^a^	Chronbach’s α	IIC^b^	ICC^c^ (95% CI) (*n* = 143)
Fatigue	9	58.56 ± 15.67	0.85	0.51–0.64	0.84 (0.78–0.88)
Cardiovascular system	3	40.29 ± 18.38	0.75	0.57–0.62	0.93 (0.91–0.95)
Digestive tract	3	43.96 ± 18.86	0.73	0.56–0.61	0.94 (0.92–0.96)
Immune system	3	51.06 ± 18.33	0.70	0.51–0.57	0.93 (0.91–0.95)
Mental status	7	55.40 ± 23.86	0.86	0.53–0.72	0.95 (0.94–0.97)
Total	25	53.67 ± 13.96	0.93	—	0.93 (0.91–0.95)

^a^mean scores (standard deviation); ^b^item-internal consistency; ^c^intraclass correlation (consistency ICC from 2-way mixed model)

#### Internal consistency

Data on the internal consistency of the subscales and composite scores are presented in Table 2. The item-subscale correlations show that each item meets the standard of 0.40 for IIC. For each item, the correlation with the subscale it was designed to represent was significantly higher than the correlations with other subscales, except for 1 item in the immune system domain: “cold intolerance” was more highly correlated with the digestive tract (0.51 vs 0.56). Internal consistency for the overall SHS was excellent (Cronbach’s α, 0.93). The Cronbach’s α value for each of the subscales was 0.7 or higher, indicating good internal consistency.

#### Factor structure

Factor analysis of SHS generally replicated our conceptualization of the 5 domains. The KMO measure of sampling adequacy was 0.93 and the Bartlett test of sphericity was significant (χ^2^(300) = 27 617.90; *P* < 0.001). Exploratory factor analysis resulted in the extraction of 5 intercorrelated factors with eigenvalues greater than or equal to 1 (Tables 3 and 4). Table 3 shows the rotated factor loadings, with factors interpreted when loadings were greater than 0.37; there was little crossloading. Based on the item structure suggested by the analysis, the factors were essentially the same as the 5 subscales. The first factor (symptoms of fatigue) shares items 1 to 6 and items 8 to 10; the second factor (symptoms of mental status) shares items 18 to 24; the third factor (symptoms of the cardiovascular system) shares items 11 to 13; the fourth factor (symptoms of the digestive tract) shares items 14 to 16; the fifth factor (symptoms of immune system) shares items 7, 17, and 25. CFA based on 5 intercorrelated factors showed a reasonable fit of the data to the factor structure: χ^2^(400) = 2517.41, *P* < 0.001; RMSEA = 0.044 (95% CI, 0.042 to 0.045); GFI = 0.914; AGFI = 0.900.

**Table 3.** Loadings of Variables on Factors (in Bold) Emerging From Factor Analysis (Maximum-Likelihood Method, Promax Rotation)

Item no.	Abbreviated item-label	Factors					Communalities

		1	2	3	4	5	
1	Exhaustion	**0.745**	−0.032	−0.028	0.022	0.049	0.560
2	Chronic fatigue	**0.606**	−0.047	0.125	0.004	0.004	0.385
3	Lethargy when working	**0.684**	0.071	−0.089	−0.060	0.055	0.487
4	Headache	**0.457**	0.065	0.050	0.001	0.127	0.232
5	Dizziness	**0.391**	0.104	0.105	0.057	0.140	0.198
6	Aching or tired eyes	**0.548**	0.022	−0.053	−0.013	0.081	0.310
7	Sore throat	0.022	−0.019	0.002	0.059	**0.533**	0.288
8	Muscle or joint stiffness	**0.832**	−0.065	−0.025	−0.026	−0.040	0.699
9	Ache in shoulder/neck/waist	**0.843**	−0.003	−0.089	−0.068	0.006	0.723
10	Heavy feeling in legs	**0.563**	0.030	0.176	−0.004	0.055	0.352
11	Breathlessness	−0.007	−0.038	**0.650**	−0.058	0.003	0.427
12	Chest congestion	0.052	0.137	**0.694**	0.225	−0.085	0.561
13	Heart palpitations	−0.009	−0.040	**0.570**	−0.058	0.006	0.330
14	Poor appetite	−0.026	−0.042	−0.042	**0.647**	0.003	0.423
15	Upset stomach	0.090	0.247	0.058	**0.636**	−0.086	0.484
16	Indigestion	−0.028	−0.041	−0.038	**0.454**	0.020	0.210
17	Cold intolerance	0.209	−0.066	−0.005	0.258	**0.387**	0.264
18	Difficulty falling asleep	−0.023	**0.472**	0.078	0.157	0.057	0.257
19	Waking up during the night	0.056	**0.510**	0.056	0.105	−0.044	0.279
20	Impaired short-term memory	0.021	**0.860**	−0.047	−0.139	−0.071	0.767
21	Inability to respond quickly	0.059	**0.830**	−0.020	−0.102	−0.069	0.708
22	Difficulty concentrating	−0.113	**0.818**	−0.012	0.010	0.057	0.685
23	Distracted for no reason	−0.014	**0.575**	0.046	0.093	0.143	0.362
24	Nervous or jittery	−0.071	**0.611**	0.019	0.141	0.172	0.428
25	Frequently catch colds	0.142	0.076	−0.045	−0.039	**0.771**	0.624

**Table 4.** Intercorrelation of Subscales (Pearson’s *r*)

Subscale	Fatigue	Cardiovascular system	Digestive tract	Immune system	Mental status
Fatigue	1.00				
Cardiovascular system	0.43^a^	1.00			
Digestive tract	0.34^a^	0.42^a^	1.00		
Immune system	0.52^a^	0.40^a^	0.40^a^	1.00	
Mental status	0.44^a^	0.32^a^	0.37^a^	0.41^a^	1.00

^a^*P* < 0.001

#### Discriminative ability

ANOVA revealed significant differences (*P* < 0.01) in total scores, and in scores for the 5 subscales, among the 3 occupation groups (Table 5). White-collar workers gave significantly higher ratings on all 5 subscales, as compared to the other 2 groups (*P* < 0.01). Blue-collar workers gave significantly higher ratings on the fatigue and cardiovascular system subscales, as compared to college students (*P* < 0.05). With respect to total score, significant differences by age group (*F*_(3,1493)_ = 16.20, *P* < 0.001) were seen among white-collar workers: the total score for those aged 18 to 30 years (52.76 ± 15.57) was significantly lower (*P* < 0.001) than those of older age groups (31–40 years, 61.23 ± 17.33; 41–50 years, 64.27 ± 19.10; 51–60 years, 62.15 ± 20.34); however, no significant differences were seen among the latter 3 groups (*P* > 0.05).

**Table 5.** SHS Scores of White-Collar Workers, Blue-Collar Workers, and College Students

Subscale	White-collar workers (*n* = 1497)	Blue-collar workers (*n* = 507)	College students (*n* = 345)	*F*_(2,2346)_	*P*
Fatigue	64.79 ± 16.64^a^	51.66 ± 15.02^b^	44.55 ± 14.23^c^	34.05	<0.001
Cardiovascular system	45.04 ± 18.55^a^	35.58 ± 15.99^b^	27.96 ± 14.28^c^	13.59	0.001
Digestive tract	44.13 ± 19.03^a^	41.09 ± 16.90	39.72 ± 16.43^c^	4.75	0.009
Immune system	55.43 ± 16.43^a^	40.91 ± 14.51	36.84 ± 14.89^c^	27.28	<0.001
Mental status	63.97 ± 18.13^a^	42.90 ± 17.96	39.32 ± 16.61^c^	29.20	<0.001

Total items	59.81 ± 18.39^a^	45.28 ± 16.66^b^	38.96 ± 14.06^c^	33.99	<0.001

*P *values are from ANOVA comparisons of the 3 groups.

^a^*P* < 0.01 for comparison between white-collar workers and blue-collar workers.

^b^*P* < 0.01 for comparison between blue-collar workers and college students.

^c^*P* < 0.01 for comparison between college students and white-collar workers.

#### Convergent validity

The correlation between the score for SHS (53.67 ± 13.96) and that for experienced stress (72.67 ± 17.12; Cronbach’s α = 0.76) was statistically significant (Pearson’s *r* = 0.57, *P* < 0.001).

### Discussion

The SHSQ-25 was shown to be reliable and valid in a large-sample health status survey in Beijing. In addition, the proportion of fully completed questionnaires (93.3%) was high, indicating that participants carefully responded to the questions.

The overall Cronbach’s α was good (0.93). When individual internal consistency was analyzed further within each domain, the α for 3 subscales (cardiovascular system, digestive tract, and immune system) was relatively low (0.70 to 0.75). This could be due to the small number of items for each subscale. However, increasing the number of items would have made the questionnaire too lengthy and participants would thus have been less likely to complete it. Temporal stability of the SHSQ-25 was excellent, as shown by the test-retest results. This indicates that the questionnaire achieved stable reliability.

One of the goals of the factor analysis was to determine whether we could extract the underlying dimensions that would support our conceptual model. The analysis resulted in 5 distinct factors, as conceptualized in our model. However, the first factor consisted of 9 items that seemed to describe 2 dimensions, including feelings of fatigue (items 1, 2, and 3) and symptoms of fatigue (items 4, 5, 6, 8, 9, and 10). Cronbach’s α of the scales was 0.74 and 0.82, respectively—lower than that for the domain of fatigue (0.85). Therefore, we did not regroup these items into 2 groups. The multidimensional structure of the instrument was further checked by CFA, which showed a good fit of the data (GFI = 0.914; AGFI = 0.900).

The questionnaire instrument was also able to discriminate between groups. As expected, there were statistically significant differences in scale scores among occupation groups, and between younger and older participants. White-collar workers had much higher SHS scores, which indicate worse health. With rapid economic development across China, increasing numbers of people with university degrees migrate to large cities to find better employment opportunities. Competition and highly demanding responsibilities are important work stressors for white-collar workers. Such employees are required to work long hours and they strive for the recognition and respect of their superiors. The fact that blue-collar workers had a higher total score and higher scores on the fatigue and cardiovascular system subscales than did college students also provides evidence of the discriminating ability of the scale. Significant differences in SHS by age were seen among white-collar workers. With increasing working age, SHS scores increased gradually, indicating worsening health. Since over half of the individuals aged over 50 years were excluded because of the results of their general medical examination, the ratings of participants aged 51 to 60 years are slightly lower than those aged 41 to 50 years. The significant correlation between SHS scores and experienced stress is evidence of the instrument’s convergent validity.

Although SHS has become a popular topic among urban Chinese, there has been no standard evaluation questionnaire. Previous tools for the assessment of SHS have not been widely adopted, and none were evaluated for reliability and validity, except for a “TCM Syndrome Questionnaire of Sub-health Status,” which comprises 124 items with 6 domains, and is based on traditional Chinese medicine.^32^ Cronbach’s α of the scale was 0.79, and the extracted 13 factors explained 48% of the variance. Although the CMI has been shown to be a valid tool for measuring general health in Chinese,^33^ it has not been widely used because the questionnaire is too lengthy and time-consuming.^31^ In contrast, questionnaire SHSQ-25 is a brief and valid instrument for the assessment of SHS.

One of the limitations of this study is that the participants were recruited at the physical examination center of Beijing Xuanwu Hospital, Capital Medical University. Hence, the sample’s demographic characteristics differ from those of city-dwelling adults in China,^7^ which might limit the generalizability of the results. In future studies it would therefore be valuable to test the questionnaire on a representative sample of urban Chinese.

## CONCLUSION

We established a valid instrument SHSQ-25 for investigating sub-health status, which is prevalent in urban Chinese. The SHSQ-25 accounts for the multidimensionality of SHS by encompassing the domains of fatigue, the cardiovascular system, the digestive tract, the immune system, and mental status. It is short and easy to complete, and therefore suitable for use in studies of the general population.

## APPENDIX

### Suboptimal health status questionnaire (SHSQ-25)

The following questions inquire about health events during the last 3 months. Answer every question by marking the appropriate box with an ‘x'. You may choose from one of the following answers:

**Table:**

1	2	3	4	5
never or almost never	occasionally	often	very often	always

**How often is it, that you (your)**		**1**	**2**	**3**	**4**	**5**
1.	were exhausted without greatly increasing your physical activity.	□	□	□	□	□

2.	fatigue could not be substantially alleviated by rest.	□	□	□	□	□

3.	were lethargic when working.	□	□	□	□	□

4.	suffered from headaches.	□	□	□	□	□

5.	suffered from dizziness.	□	□	□	□	□

6.	eyes ached or were tired.	□	□	□	□	□

7.	suffered from a sore throat.	□	□	□	□	□

8.	muscles or joints felt stiff.	□	□	□	□	□

9.	have pain in your shoulder/neck/waist.	□	□	□	□	□

10.	have a heavy feeling in your legs when walking.	□	□	□	□	□

11.	feel out of breath while sitting still.	□	□	□	□	□

12.	suffered from chest congestion.	□	□	□	□	□

13.	were bothered by heart palpitations.	□	□	□	□	□

14.	appetite is poor.	□	□	□	□	□

15.	suffered from heartburn.	□	□	□	□	□

16.	suffered from nausea.	□	□	□	□	□

17.	could not tolerate the cold.	□	□	□	□	□

18.	had difficulty falling asleep.	□	□	□	□	□

19.	had trouble with waking up during night.	□	□	□	□	□

20.	had trouble with your short-term memory.	□	□	□	□	□

21.	could not respond quickly.	□	□	□	□	□

22.	had difficulty concentrating.	□	□	□	□	□

23.	were distracted for no reason.	□	□	□	□	□

24.	felt nervous or jittery.	□	□	□	□	□

25.	caught a cold in the past 3 months.	□	□	□	□	□
